# Identification of potential pathogenic genes for lung adenocarcinoma induced by 6PPD-quinone through network toxicology and SHAP analysis

**DOI:** 10.3389/ftox.2026.1948595

**Published:** 2026-10-19

**Authors:** Xiang Xiong, Jian Ding, Wen Liu, Baolan Li, Chuan Yao

**Affiliations:** 1 Department of Cardiothoracic Surgery, The Affiliated Hospital of Jiujiang University, Jiujiang, Jiangxi, China; 2 Tianjin Medical University, Tianjin, China

**Keywords:** 6PPD-quinone, lung adenocarcinoma, molecular docking, network toxicology, SHAP analysis

## Abstract

**Background:**

Lung cancer continues to maintain a high mortality rate, with lung adenocarcinoma (LUAD) being its most common type. As an emerging environmental pollutant, 6PPD-quinone is attracting increasing attention. Through network toxicology, we constructed a prognostic model and validated it with molecular docking and dynamics simulations, revealing its underlying molecular mechanisms.

**Methods:**

RNA-seq and clinical data of LUAD were obtained from the TCGA database, and differential expression analysis was performed using DESeq2. Putative targets of 6PPD-quinone were retrieved from DIGEP-Pred 2.0 and SEA databases, while LUAD-associated genes were collected from GeneCards, CTD, and OMIM. Overlapping genes with DEGs were identified as potential targets. Protein–protein interaction networks were constructed via STRING and visualized with Cytoscape. The hub genes were screened using four algorithms (Degree, EPC, MCC, MNC). GO and KEGG enrichment analyses were conducted on both potential and hub genes. A prognostic model was built based on Cox regression, with risk scores calculated for survival and independent prognostic analyses. SHAP analysis was applied to interpret feature contributions. Molecular docking and molecular dynamics simulations assessed binding stability between 6PPD-quinone and key genes, and qPCR validated expression changes in BEAS-2B and A549 cells with or without 6PPD-quinone exposure.

**Results:**

6PPD-quinone-linked LUAD were used to identify 31 potential targets and screen 18 network hub genes. Through a seven-gene prognostic signature (GPI, FAM83D, KRT17, CTLA4, MAP3K8, SIX1, ABCC2) construction, clear stratification into high- and low-risk groups was achieved with significantly different survival rates (1-year AUC = 0.711). SHAP analysis highlighted GPI, FAM83D, KRT17, and ABCC2 as major pathogenic contributors. Molecular docking demonstrated stable binding between 6PPD-quinone and GPI, FAM83D, and ABCC2 (binding energies < −5 kcal/mol). Furthermore, MD simulations confirmed the stability of the ABCC2-ligand complex. qPCR validation confirmed that exposure to 6PPD-quinone markedly enhanced the expression of these genes in A549 cells.

**Conclusion:**

This study identifies GPI, FAM83D, and ABCC2 as potential pathogenic genes linking 6PPD-quinone exposure to LUAD, possibly through genetic dysregulation. The integrative network toxicology and SHAP-based approach provide a robust framework for uncovering environmental carcinogenesis mechanisms, offering new insights into the health risks of tire-derived pollutants.

## Introduction

Lung cancer is the type of cancer that exhibits the highest rates of occurrence and mortality (Song et al., 2024). It is histologically categorized into two main types: non-small cell lung cancer (NSCLC) and small cell lung cancer, with NSCLC comprising roughly 80% of all diagnosed instances (Zhang et al., 2025; Yu et al., 2025). Among these, lung adenocarcinoma (LUAD) stands out as the most common subtype, accounting for about 40% of cases. Recent figures indicate that in 2022, there were around 2,480,308 new diagnoses and 1,817,131 fatalities (Hyman et al., 2025). Early screening can identify numerous instances of lung cancer in its initial stages, the majority of patients displaying clinical symptoms typically receive a diagnosis at a more advanced phase, thereby missing the chance for surgical intervention. Despite the considerable advancements achieved through chemotherapy, immunotherapy, and targeted therapies, significant challenges still lie ahead (Di Federico et al., 2025; Lin et al., 2025; Shen et al., 2025). Particularly, understanding the fundamental mechanisms of LUAD, along with the potential uncontrollable factors in our environment, continues to be vital challenges that require attention.

For many years, environmental pollutants have been recognized as significant contributors to lung cancer, with 6PPD-quinone, a derivative of the additive *N*-(1,3-dimethylbutyl)-*N*′-phenyl-*p*-phenylenediamine (6PPD) found in tire rubber, garnering increasing attention (Kazmi et al., 2024). Tire particles containing 6PPD-quinone can infiltrate rivers, lakes, and other water bodies via rainwater, leading to multi-organ damage affecting the human liver, lungs, intestines, nervous system, and reproductive system (Tang et al., 2025; Wang Z. et al., 2026; Zhu and Xu, 2025; Zuo et al., 2026). Recent studies have demonstrated that 6PPD-quinone exhibits reproductive toxicity by impairing sperm quality in mice, thereby compromising fertility (Yao et al., 2024). Moreover, pregnant mice exposed to 6PPD-quinone displayed enhanced embryo transfer capacity, indicating its potential toxicity within the reproductive system (Zhao et al., 2023). Li et al. elucidated the potential molecular mechanisms underlying 6PPD-quinone-induced respiratory inflammation through network toxicology approaches (Li et al., 2025). Additionally, research has confirmed that 6PPD-quinone promotes lung tissue inflammation and fibrosis through interactions with molecules such as Ripk1, Fadd, and Smad2 (He et al., 2024). In A549 cells, a human lung adenocarcinoma cell line, 6PPD-quinone induces DNA fragmentation via oxidative stress and other pathways, which correlates with increased intracellular levels of reactive oxygen species (ROS) and 8-hydroxy-2′-deoxyguanosine (8-OHdG) (Hyman et al., 2025; Damdam et al., 2026).

In summary, 6PPD-quinone, an emerging environmental pollutant, is closely related to our daily lives. Previous studies have indicated that 6PPD-quinone exhibits potential pulmonary toxicity. However, the underlying pathogenic mechanisms through which 6PPD-quinone contributes to LUAD remain unclear. This study aims to explore these molecular mechanisms and provide new insights into urban air pollution.

## Methods

### Data acquisition from TCGA database

The RNA-sequencing (RNA-seq) data and clinical information for 59 normal individuals and 542 LUAD patients were gathered from The Cancer Genome Atlas (TCGA) database (https://portal.gdc.cancer.gov/). Samples that had unclear staging or survival data were removed from the analysis. The DESeq2 package was employed to conduct the differential expression analysis, applying criteria for the identification of differential expression genes (DEGs) with an adjusted p-value of less than 0.05 and a |log2FoldChange| exceeding 1 (Xiong et al., 2025).

### Collection of 6PPD-quinone and LUAD related genes

Retrieve the three-dimensional structure and the Canonical SMILES notation for the compound 6PPD-quinone from the database (https://pubchem.ncbi.nlm.nih.gov/). Potential human target genes influenced by 6PPD-quinone were gathered from the DIGEP-Pred 2.0 (Ivanov et al., 2024), and the Similarity Ensemble Approach database (https://sea.bkslab.org/). Genes associated with LUAD were sourced from the GeneCards database (https://www.genecards.org/), the Comparative Toxicogenomics Database (https://ctdbase.org/), and the OMIM database (https://omim.org/) using the term “lung adenocarcinoma.” The combined gene set of 6PPD-quinone and LUAD was determined using the “ggvenn” package. Ultimately, potential genes through which the compound influences the disease were identified by intersecting the target genes for 6PPD-quinone, LUAD-associated genes, and differentially expressed genes from the TCGA database.

### Construction of PPI network

An analysis of protein-protein interactions (PPI) was performed on the overlapping genes found within 6PPD-quinone targets, LUAD targets, and differentially expressed genes, utilizing the STRING database (https://cn.string-db.org/) specifically configured for “*Homo sapiens*”. A “Medium Confidence” threshold, with an interaction score exceeding 0.4, was employed. The outcomes from STRING were subsequently imported into Cytoscape software to assess the topological characteristics of the PPI network.

### Identification of network hub genes

In Cytoscape, the MCODE plugin was employed to pinpoint the most significant module within the PPI network. Furthermore, the cytoHubba plugin was used to calculate “Node Scores” through nine different algorithms, including Betweenness, Closeness, Degree, DMNC, EPC, MCC, MNC, Radiality, and Stress. For each of these algorithms, the top twenty genes exhibiting the highest scores were identified as crucial genes. Following this, we conducted an intersection analysis on the key genes revealed by four specific algorithms (Degree, EPC, MCC, MNC), categorizing the genes found in all intersections as “hub genes”.

### Analysis of functional enrichment

Enrichment analyses for potential or hub genes were performed with the clusterProfiler package to explore Gene Ontology (GO) terms, which include biological processes (BP), molecular functions (MF), and cellular components (CC), in addition to Kyoto Encyclopedia of Genes and Genomes (KEGG) pathways. The identification of significantly enriched terms or pathways was based on an adjusted p-value threshold of <0.05, applying the Benjamini-Hochberg method for correction.

### Identification of prognostic genes and construction of prognostic models

Utilizing the identified hub genes, expression data from TCGA alongside survival data were integrated. Prognosis-associated genes were identified through Cox analysis (with filtering criteria set at P-value <0.05) and were visualized in forest plots. A prognostic model was developed employing the prognosis-associated genes to formulate the model equation, after which the risk score was computed using the equation: Risk score = β gene1 × Exp gene1 + β gene2 × Exp gene2 + β gene3 × Exp gene3 + • + β genen × Exp genen.

### Survival analysis and independent prognostic analysis

A formula was developed by this model to determine the risk score associated with each sample. Based on the median risk score, the samples were divided into two groups: high-risk and low-risk. The “survival” package was employed to analyze the overall survival (OS) and progression-free survival (PFS) among LUAD patients in these designated groups. Multivariate analyses for independent prognostic factors were performed to evaluate the prognostic significance of clinical characteristics. Risk scores were used to create a visualization that depicts the survival duration of LUAD patients and the expression levels of lncRNAs through the “pheatmap” package. Moreover, the area under the ROC curve (AUC) for clinical features at 1, 3, and 5 years was calculated for the training, testing, and combined groups using the “survival ROC” package.

### Model interpretation

Due to the fundamental “black-box” characteristic of machine learning models, we utilized the SHapley Additive exPlanations (SHAP) algorithm to evaluate the contribution of every feature to the predictive results. This method allocates a SHAP value to every feature, facilitating a clear understanding of its impact on the predictions made by the model.

### Molecular docking analysis

In order to enhance our understanding of the intermolecular interactions between 6PPD-quinone and the hub proteins identified in this study, we employed a molecular docking approach. We obtained ligand structures in SDF format and the 3D models of core target proteins from PubChem and UniProt, respectively. Prior to docking, we prepared the protein structures by eliminating water molecules and incorporating hydrogen atoms, while optimizing the geometry of the ligands via the MMFF force field. For the docking process, we conducted a blind docking procedure by inputting the 3D structural data of the target compounds and proteins. All docking operations were carried out using AutoDock Vina, and the results were subsequently analyzed and visualized through structure-based blind docking techniques (Liu et al., 2022; Yang et al., 2022).

### Molecular dynamics simulation analysis

Molecular dynamics (MD) simulations were conducted on the protein-ligand complex using the GROMACS 2025.3 software package (Páll et al., 2020). The system was organized within a periodic cubic boundary box and was solvated with the AMBER14SB force field along with the TIP3P water model (Maier et al., 2015). The AMBER14SB force field was obtained from the standard GROMACS distribution (ff14SB, as implemented in the AmberTools package). It was chosen because it offers well-balanced accuracy for protein backbone and side-chain conformations and has been extensively validated with the TIP3P water model, which we used. The molecular topology file for the small molecules was generated utilizing the Sobtop software (Tian Lu, Sobtop, Version [1.0(dev5)], http://sobereva.com/soft/Sobtop (accessed on 6 January 2026), followed by the addition of ions to achieve a physiological concentration of 150 mM NaCl. For the small-molecule ligand (6PPD-quinone), partial charges were derived using the AM1-BCC charge model via the Sobtop software, which is a widely accepted semi-empirical method for generating reasonable electrostatic potentials for drug-like molecules. The simulation proceeded with 500 steps of energy minimization, employing the conjugate gradient algorithm (convergence tolerance 100.0 kJ/mol·nm, step size 0.01 nm), succeeded by a restrained dynamics simulation of 100 ps (time step 1 fs) and a 100 ns formal simulation (time step 2 fs). The temperature was controlled at 298.15 K using the V-rescale method (time constant 0.2 ps), while the pressure was regulated at 1.0 bar through the C-rescale method (time constant 0.5 ps/2.0 ps). The simulations applied LINCS constraints for hydrogen bonds, PME for long-range electrostatics (cutoff radius 1.0 nm), and a Verlet cutoff method for van der Waals interactions (cutoff radius 1.0 nm), enabling energy and pressure dispersion corrections. During the simulation, the motion of the system’s center of mass was eliminated, and angular momentum removal along with independent temperature coupling was implemented for the protein-ligand complex during the production runs. Analyses performed comprised the root-mean-square deviation (RMSD) of the Cα atoms in the protein backbone, the radius of gyration (Rg), the root-mean-square fluctuation (RMSF) of the residues, the solvent accessible surface area (SASA), and the count of hydrogen bonds between the protein and ligand. We additionally created two-dimensional (2D) and three-dimensional (3D) free energy landscape (FEL) maps to illustrate the conformational stability of the complex. These landscape maps utilize Rg and RMSD as coordinates to depict the distribution of free energy throughout the conformational space. The regions marked in dark blue represent the global free energy minima, which are associated with the most thermodynamically stable conformations.

### Cell culture and treatment

BEAS-2B human normal bronchial epithelial cells and A549 human lung adenocarcinoma cells were purchased from Fuheng Biotechnology (Shanghai, China). Both types of cells were cultured in DMEM medium containing 10% fetal bovine serum and maintained in a constant temperature incubator at 37 °C with 5% CO_2_. BEAS-2B cells were used as normal controls without any treatment. A549 cells were divided into an untreated group and a treatment group (A549 + 6PPD-quinone). The latter was treated with 40 ng/mL 6PPD-quinone (MCE, purity ≥98%) continuously for 48 h.

### qPCR analysis

Using TRIzol reagent (Invitrogen), total RNA was extracted from BEAS-2B, A549, A549 treated with 6PPD-quinone (40 ng/mL, 48 h) (Hyman et al., 2025; Damdam et al., 2026). The concentration and purity of RNA was measured using a NanoDrop 2000. 1 μg of total RNA was reverse transcribed using PrimeScript RT Master Mix (Takara). qPCR was performed in triplicate in 20 μL reactions on a LightCycler 480 system using SYBR Green Master Mix (Roche), 0.25 μM of each primer, and 2 μL of cDNA. The conditions for thermal cycling were 95 °C for 5 min, 40 cycles at 95 °C for 10 s, then 60 °C for 20 s, and finally 72 °C for 20 s. Melting curve analysis was utilized to ensure amplification specificity. As marked in Supplementary Table S1, the primer sequences were purchased from the OriGene Biotechnology Company (Beijing, China). In addition, the relative gene expression was calculated by using the 2^−ΔΔCT^ method by taking GAPDH as the internal control and BEAS-2B as the calibrator.

### Statistical analysis

Statistical analyses were conducted using R software (version 4.5.1) for this study. The data are expressed as mean ± SD derived from three separate experiments. Group differences were assessed via one-way ANOVA, followed by Tukey’s post-hoc test for further evaluation. A p-value of less than 0.05 was regarded as statistically significant.

## Results

### Identification of potential targets

The flowchart is shown in Figure 1. RNA-seq data was collected from 542 primary tumor tissues of LUAD and 59 solid normal tissues sourced from the TCGA database. The differential analysis conducted between the tumor and normal groups revealed a total of 3634 genes that were differentially expressed, which included 2,092 genes that were upregulated and 1,542 genes that were downregulated (Figure 2A). From the DIGEP-Pred 2.0, and Similarity Ensemble Approach databases, we identified 519 human target genes related to 6PPD-quinone. Furthermore, we obtained 8,336 genes associated with LUAD from the GeneCards, Comparative Toxicogenomics, and OMIM databases (Supplementary Figure S1). By conducting a cross-analysis of differentially expressed genes from LUAD patients in the TCGA database, predicted target genes for 6PPD-quinone, and genes linked to LUAD, we discovered 31 potential targets that may be associated with LUAD induced by 6PPD-quinone (Figure 2B).

**FIGURE 1:**
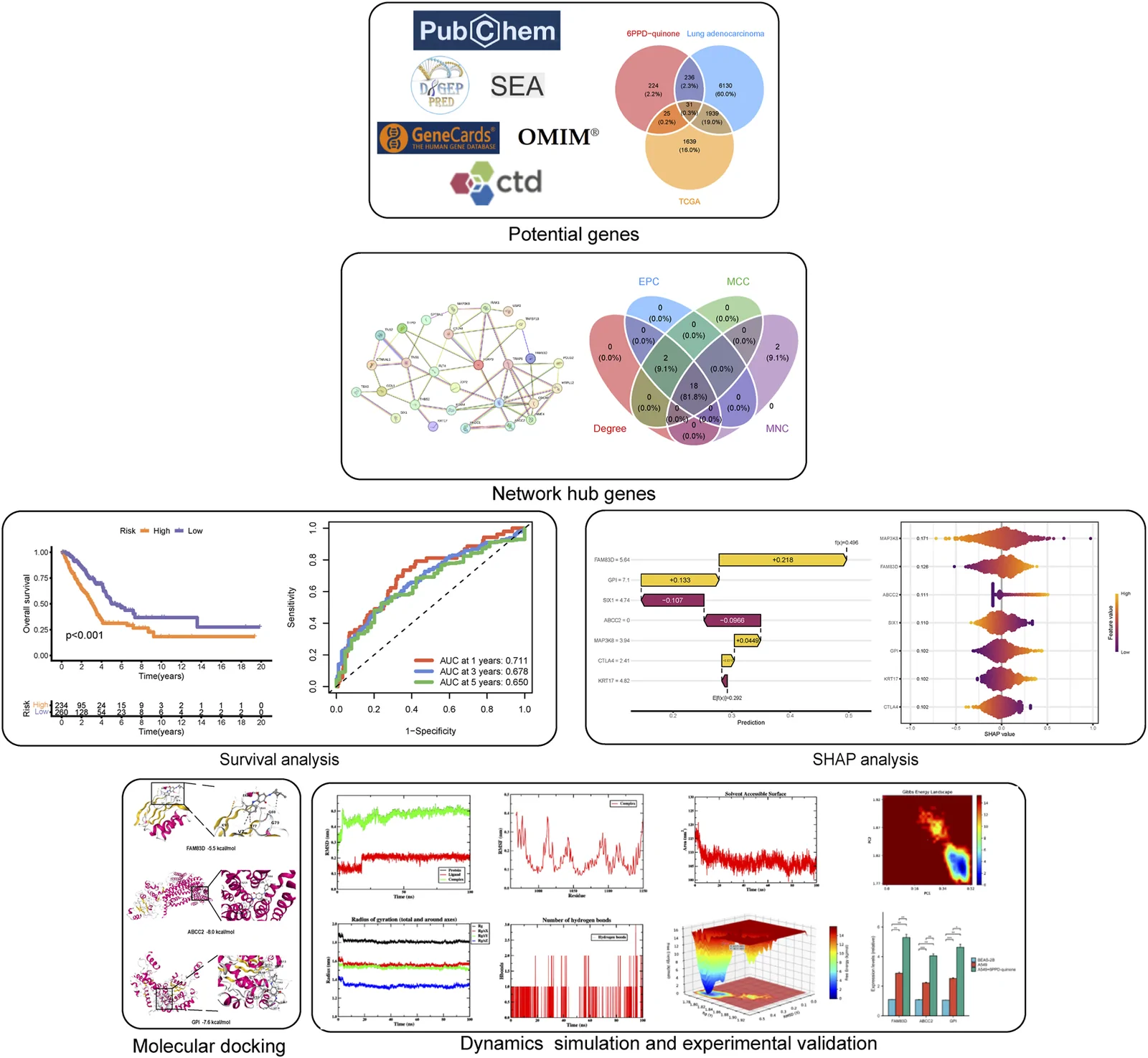
The flowchart outlines that we identified the network hub genes of 6PPD-quinone-related lung adenocarcinoma through the TCGA database and network toxicology analysis, constructed a prognostic model, and validated it through survival analysis, SHAP analysis, molecular docking, dynamics simulation, and experimental verification.

**FIGURE 2:**
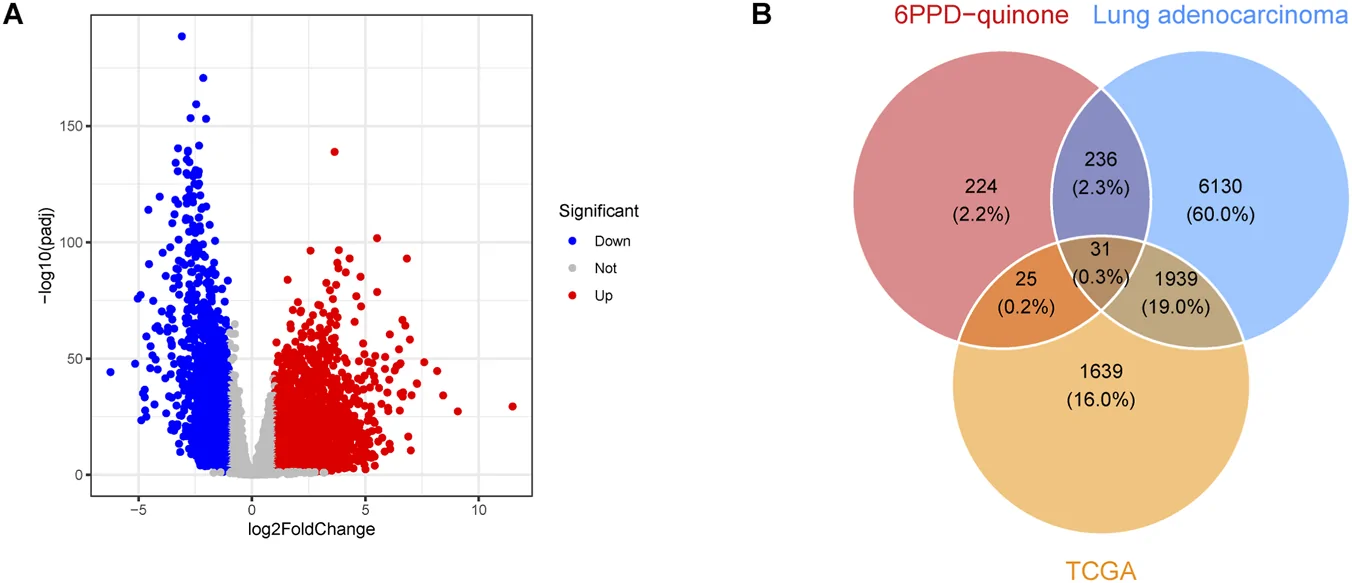
Identification of potential genes. **(A)** Volcano plot shows DEGs based on logFC and significance. Red dots are upregulated, blue dots are downregulated, and grey dots are non-significant. **(B)** The Venn diagram displays the intersecting genes (brown) among 6PPD-quinone (red), lung adenocarcinoma (blue), and TCGA (orange).

### Enrichment analysis of potential targets

Enrichment analysis was conducted on 31 potential targets, filtered with an adjusted P-value of less than 0.05. Regarding the BP terms, the three most enriched processes identified were “mesenchymal cell differentiation,” “regulation of immunoglobulin production,” and “immunoglobulin production.” For the CC terms, the leading enriched components included “mitochondrial intermembrane space,” “intercellular bridge,” and “organelle envelope lumen.” In the context of MF terms, the three primary functions were “cytokine activity,” “DNA−binding transcription repressor activity, RNA polymerase II−specific,” and “DNA−binding transcription repressor activity” (Figure 3A). In the KEGG pathway analysis, the three most enriched pathways were “Starch and sucrose metabolism,” “Biosynthesis of nucleotide sugars,” and “Amino sugar and nucleotide sugar metabolism” (Figure 3B).

**FIGURE 3:**
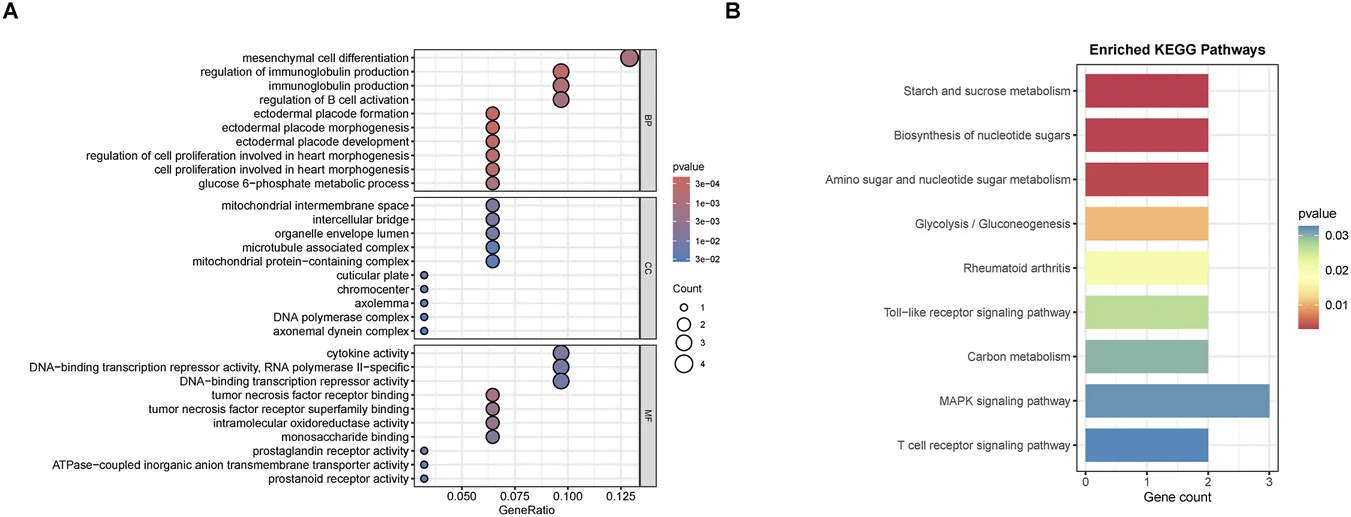
Enrichment analysis results of 31 potential genes. The bubble plots and bar chart respectively display the enrichment results of GO **(A)** and KEGG **(B)**. The vertical axis represents the names of GO and KEGG, the horizontal axis represents the proportion and count of genes. The size of the circles indicates the number of genes enriched in each GO, and the colors of the bubbles and bar charts represent the magnitude of the p-value, with darker colors indicating smaller p-values.

### Protein-protein interaction

In order to delve deeper into the interactions of the 31 potential genes, we created a protein-protein interaction network (Figure 4A). In this visualization, different genes are depicted as nodes, and the connections between them illustrate the various interaction relationships of the proteins. Utilizing Cytoscape, we visualized this network, with nodes that are darker in color indicating a higher likelihood of being hub genes within the network (Figure 4B).

**FIGURE 4:**
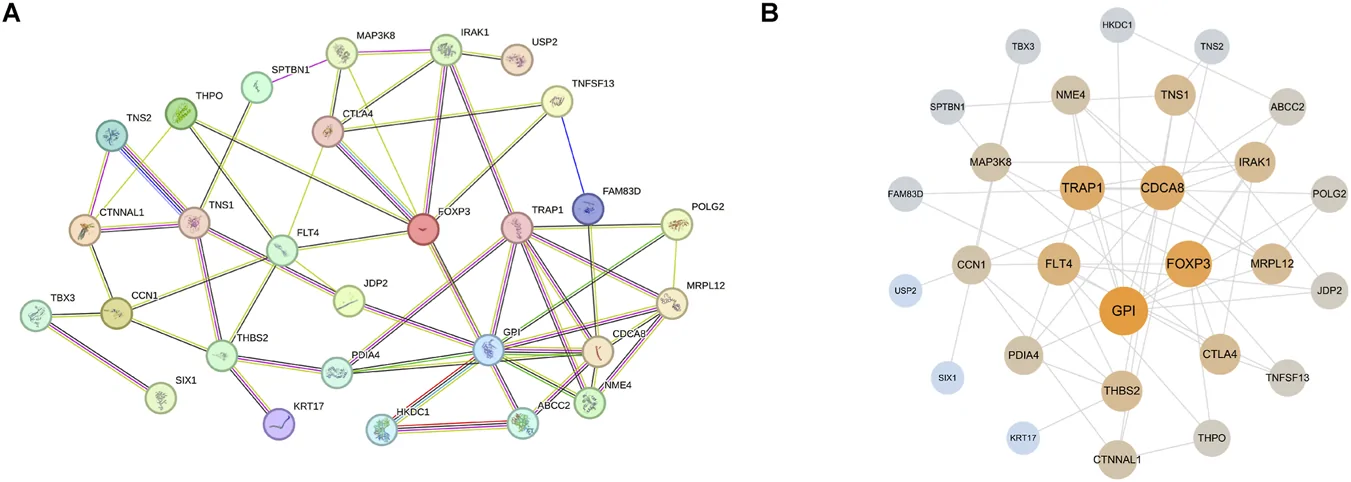
The interaction relationships between different proteins. **(A)** Proteins are connected by lines of different colors, representing various interaction relationships between them. Green represents evidence of gene neighborhood; red represents evidence of gene fusions; blue represents evidence of gene co-occurrence. **(B)** The redder a node is, the more connections it has, indicating that the node is more likely to be a hub gene in the network.

### Identification of network hub genes and enrichment analysis

We selected the 20 genes with the highest scores using four different methods (EPC, MCC, Degree, and MNC) as the hub genes for the network. The intersection of the hub genes identified by these four approaches resulted in 18 final hub genes that were used for further analysis (Figure 5A). Regarding the BP terms, the three most enriched processes identified were “regulation of immunoglobulin production,” “immunoglobulin production,” and “regulation of B cell activation.” For the CC terms, the leading enriched components included “mitochondrial intermembrane space,” “organelle envelope lumen,” and “mitochondrial protein−containing complex.” In the context of MF terms, the three primary functions were “tumor necrosis factor receptor binding,” “tumor necrosis factor receptor superfamily binding,” and “intramolecular oxidoreductase activity.” In the KEGG pathway analysis, the three most enriched pathways were “Rheumatoid arthritis,” “MAPK signaling pathway,” and “Toll−like receptor signaling pathway “ (Figure 5B).

**FIGURE 5:**
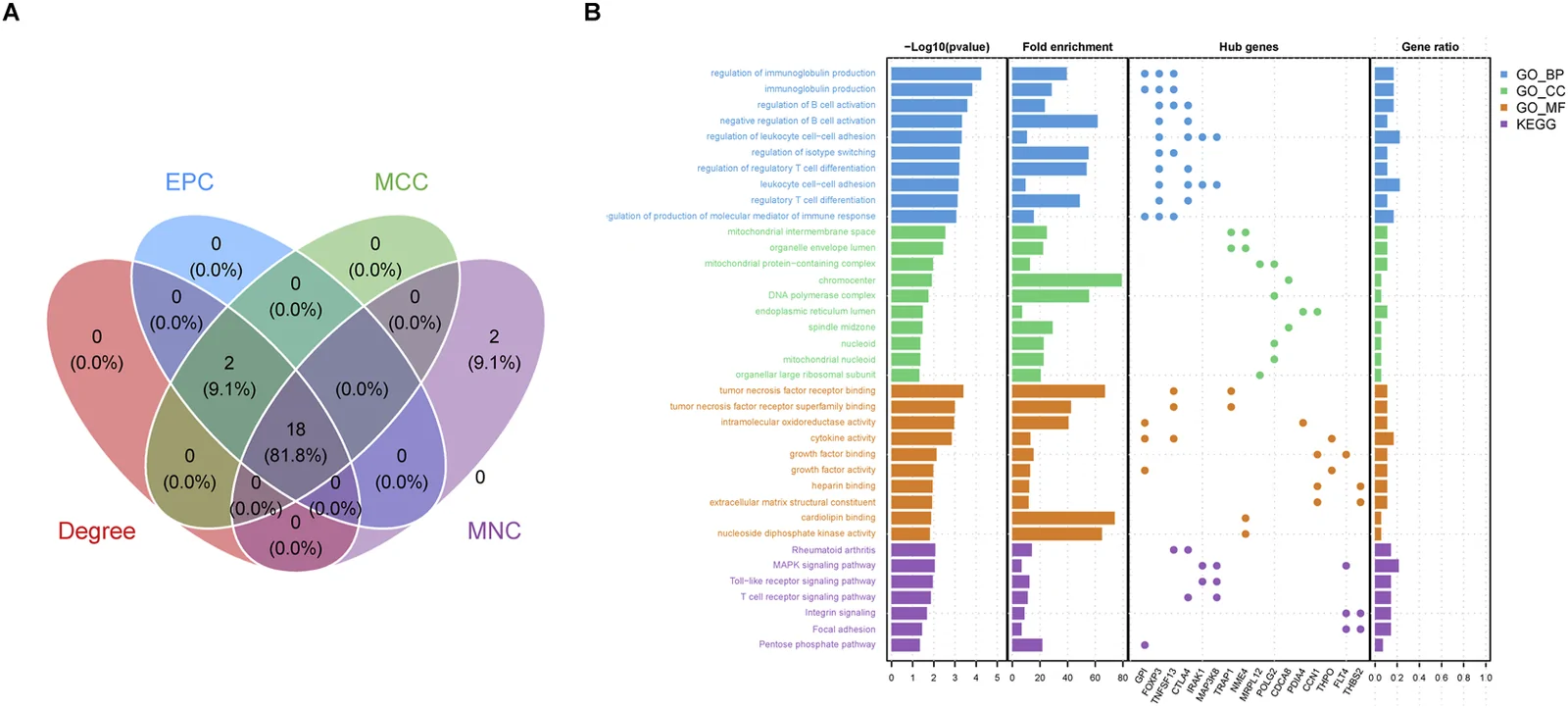
We identified 18 network hub genes. **(A)** The intersection of the network was obtained using four scoring methods: EPC, MCC, Degree, and MNC. **(B)** The GO and KEGG analysis results of the network hub genes.

### Construction of prognostic models and SHAP analysis

Cox analysis was conducted on the hub genes, focusing on those related to prognosis, and the findings were visualized using a forest plot (Supplementary Figure S2). In this visualization, high-risk genes are represented in orange, while low-risk genes are indicated in purple-orange. Our prognostic model was developed based on prognostic genes, from which a risk score was derived using these three hub genes. The formula for calculating the risk score is expressed as follows: Risk score = (0.198004277884128 * GPI) + (0.13063072524661 * FAM83D) + (0.0532905987544607 * KRT17) + (−0.122143817320388 * CTLA4) + (−0.295959888111643 * MAP3K8) + (−0.0933967084814659 * SIX1) + (0.0783409163682808 * ABCC2). To enhance our comprehension of the decision-making process of the prognostic model, we employed SHAP analysis for interpretation. By arranging the genes according to their contributions to the model, we could identify those with the most notable influence. The waterfall plot illustrated the predictive performance of individual genes, revealing that the average probability predicted by the model for all samples is 0.292, while the final prediction for this particular sample is 0.496 (Figure 6A). Additionally, analysis using the bee swarm plot demonstrated that MAP3K8 (0.171) has the highest SHAP value, while CTLA4 (0.102) has the lowest SHAP value. Among them, as the gene expression value increases, the SHAP values of FAM83D (0.128), ABCC2 (0.111), GPI (0.102), and KRT17 (0.102) also increase (Figure 6B).

**FIGURE 6:**
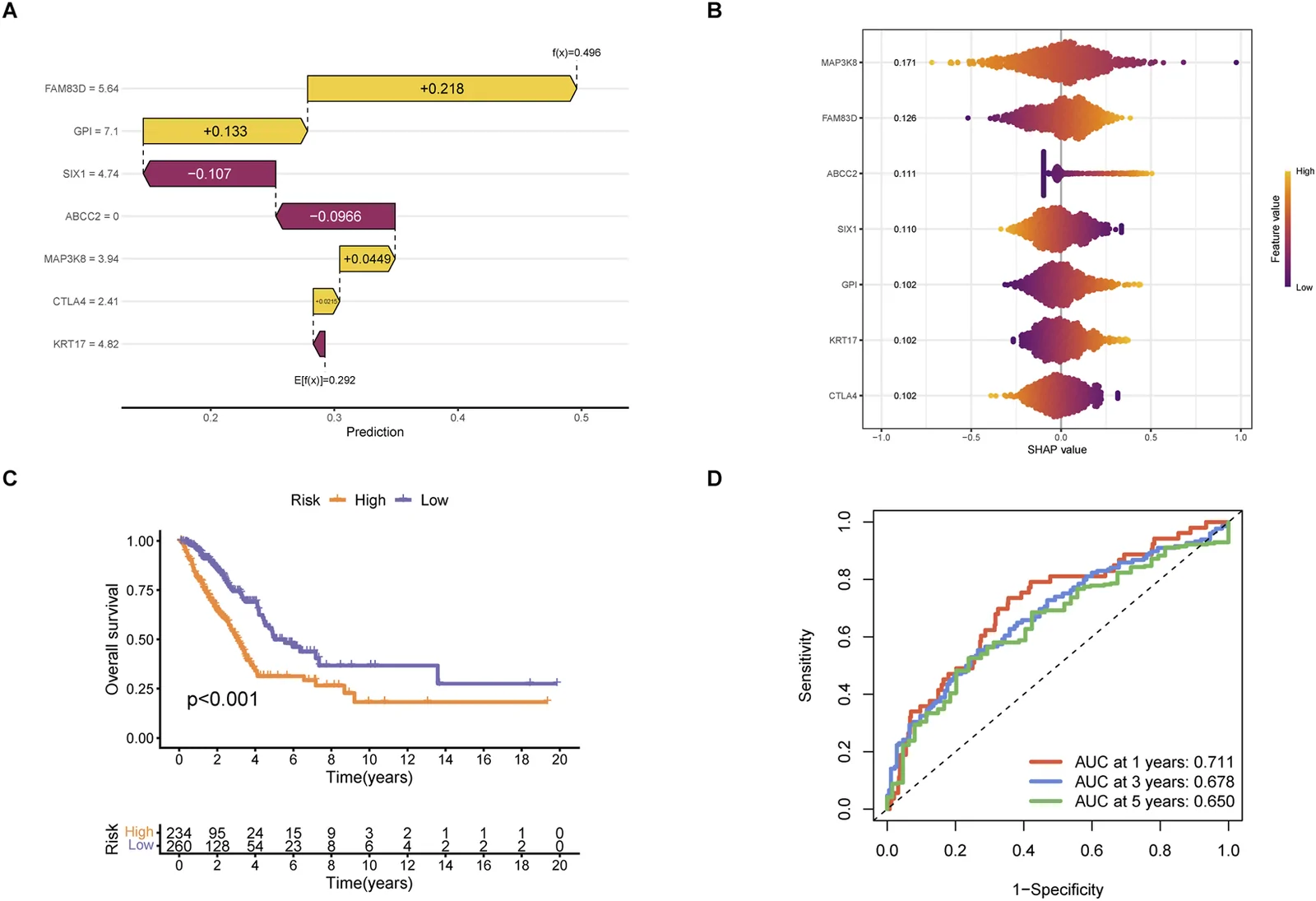
Prognostic analysis results of hub genes. SHAP analysis results are displayed through **(A)** waterfall plot and **(B)** bee swarm plot. **(C)** Prognostic survival analysis. **(D)** ROC curve.

### Survival and independent prognostic analysis

The analysis of survival curves indicated a significant survival difference between high-risk and low-risk groups, reinforcing the model’s capability to discriminate effectively between these risk categories (Figure 6C). The ROC curve analysis showed AUC values of 0.711, 0.678, and 0.650 for overall survival rates at 1, 3, and 5 years, respectively, indicating the model’s proficiency in predicting patient survival time (Figure 6D). The examination of the risk curve showed that patients categorized in the low-risk group experienced a significantly lower mortality rate than those in the high-risk group. Furthermore, a heatmap illustrated the varying levels of risk for three hub genes, with CTLA4, MAP3K8, and SIX1 identified as low-risk genes, while GPI, FAM83D, KRT17, and ABCC2 were classified as a high-risk gene (Figure 7A). This is consistent with the analysis results we observed from the SHAP model. The multivariate Cox regression analysis indicated that stage II (HR = 2.049, 95% CI 1.403–2.99; P < 0.001), stage III (HR = 2.818, 95% CI 1.894–4.19; P < 0.001), stage IV (HR = 3.554, 95% CI 2.016–6.27; P < 0.001), and a high risk score (HR = 1.848, 95% CI 1.346–2.54; P < 0.001) were identified as independent risk factors for overall survival (Figure 7B). This highlights the significance of the risk signature’s prognostic value independent of other variables.

**FIGURE 7:**
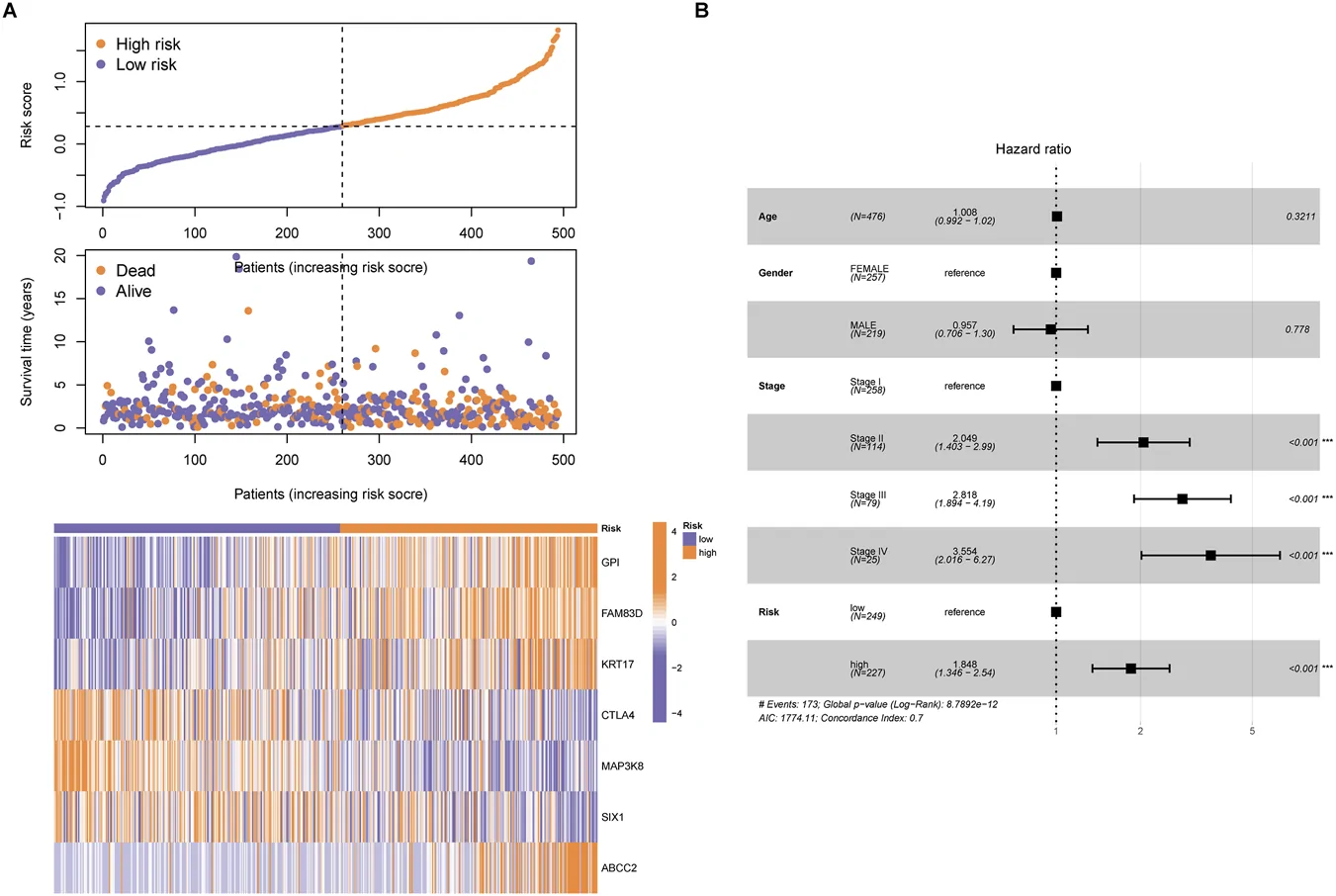
Identify independent prognostic factors. **(A)** Construct risk curves based on high- and low-risk groups. **(B)** The forest plot displays the results of independent prognostic analysis.

### Molecular docking

Based on the contributions of each gene in the SHAP analysis and the results of the risk heatmap, we selected GPI, FAM83D, and ABCC2 for subsequent molecular docking analysis. To delve deeper into the binding characteristics of the three hub genes associated with prognosis and 6PPD-quinone, we carried out a molecular docking analysis to assess their binding stability. The findings indicated that the binding energies for FAM83D (−5.5 kcal/mol), ABCC2 (−8.0 kcal/mol), and GPI (−7.6 kcal/mol) were all negative, which suggests that these molecules can bind with the compound spontaneously without the need for external energy. Additionally, each of the three molecules displayed binding energies below −5 kcal/mol, which points to considerable binding affinities. We utilized AutoDock Vina for docking simulations and made visual adjustments to facilitate a clearer observation of their docking configurations (Figures 8A–C). These outcomes highlight a significant relationship between 6PPD-quinone and the aforementioned hub genes.

**FIGURE 8:**
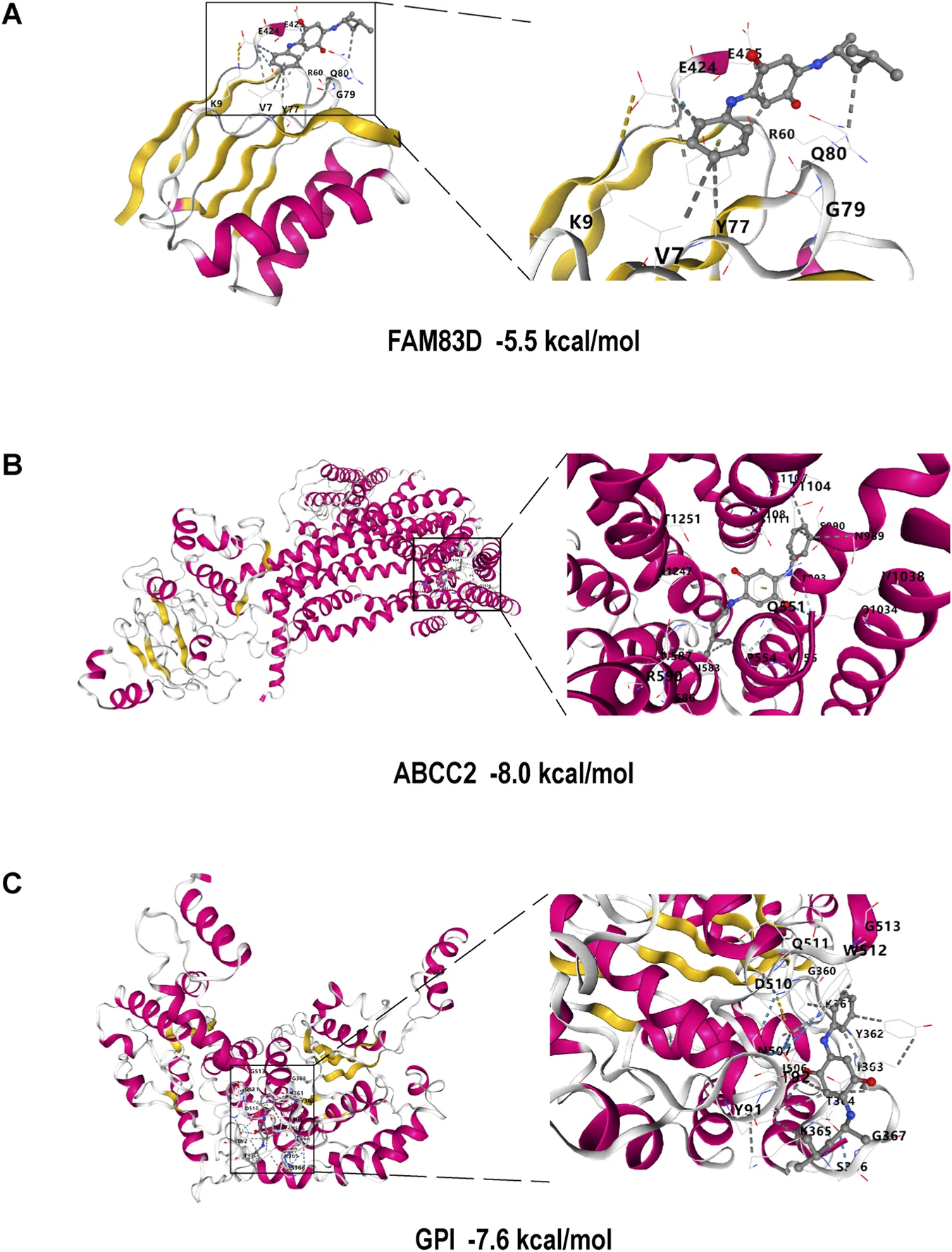
Molecular docking results of **(A)** FAM83D, **(B)** ABCC2 and **(C)** GPI. Their molecular binding energies are −5.5 kcal/mol, −8.0 kcal/mol, and −7.6 kcal/mol, respectively.

### Molecular dynamics simulation and qPCR validation

The molecular docking results indicated that ABCC2 had a higher binding energy compared to other pathogenic genes and may be a potential binding target. Through molecular dynamics simulations and experimental validation, we have further understood the relationship between 6PPD-quinone and potential disease-causing genes. RMSD quantifies how much two conformations differ from one another. A lower RMSD value signifies enhanced structural similarity, indicating reduced conformational deviations, which can be useful in evaluating system stability. Conversely, RMSF assesses the average degree of fluctuation for a particular atom or residue over a given timeframe. A higher RMSF value implies that the atom exhibits more pronounced motion (greater flexibility). In Figure 9A, it can be observed that the RMSD of the protein, ligand, and complex overall fluctuates within the range of 0.1–0.5 nm. There is no significant drift within 100 ns, indicating that the system has reached equilibrium and remains stable during the simulation. Figure 9B shows that the RMSF values of the complex remain relatively low, mainly below 0.3 nm, indicating that the structures of these residues are stable. Rg can be used to describe overall structural changes and characterize the compactness of protein structures, with larger Rg variations indicating greater system expansion. The overall Rg stabilized at around 1.8 nm, and the Rg along each axis also remained stable, as shown in Figure 9C. The dynamics of hydrogen bonds formed between the small molecules and the target protein throughout the kinetic process are detailed in Figure 9D, where the hydrogen bond counts range from 0 to 3, with an estimate of about one bond present in the majority of instances. This suggests that effective hydrogen bond interactions exist between the ligand and the target protein. SASA is a metric utilized to evaluate the surface area of proteins. The simulation provided insights into the SASA between the target protein and the small molecules (Figure 9E), demonstrating minimal fluctuations in SASA post receptor-ligand interaction, which indicates that the binding of the ligand has a negligible effect on the protein structure. Furthermore, FEL analysis reveals that the complex system showcases a significant free energy minimum region (depicted by dark blue basins in the figure) within designated ranges, highlighting that this conformational state results in optimal thermodynamic stability for the system (Figures 9F,G). To validate the pathogenic genes identified by network toxicology and SHAP analysis, we examined the mRNA expression levels of FAM83D, ABCC2, and GPI in BEAS-2B, A549, and A549 treated with 6PPD-quinone (40 ng/mL, 48 h) using qPCR. As shown in Figure 9H, the expression levels of all three genes were significantly higher in A549 cells compared with BEAS-2B cells. In A549 cells treated with 6PPD-quinone, we observed that the expression levels of GPI, FAM83D, and ABCC2 were significantly upregulated. The same result was also found in comparison with BEAS-2B cells, which is consistent with our initial hypothesis.

**FIGURE 9:**
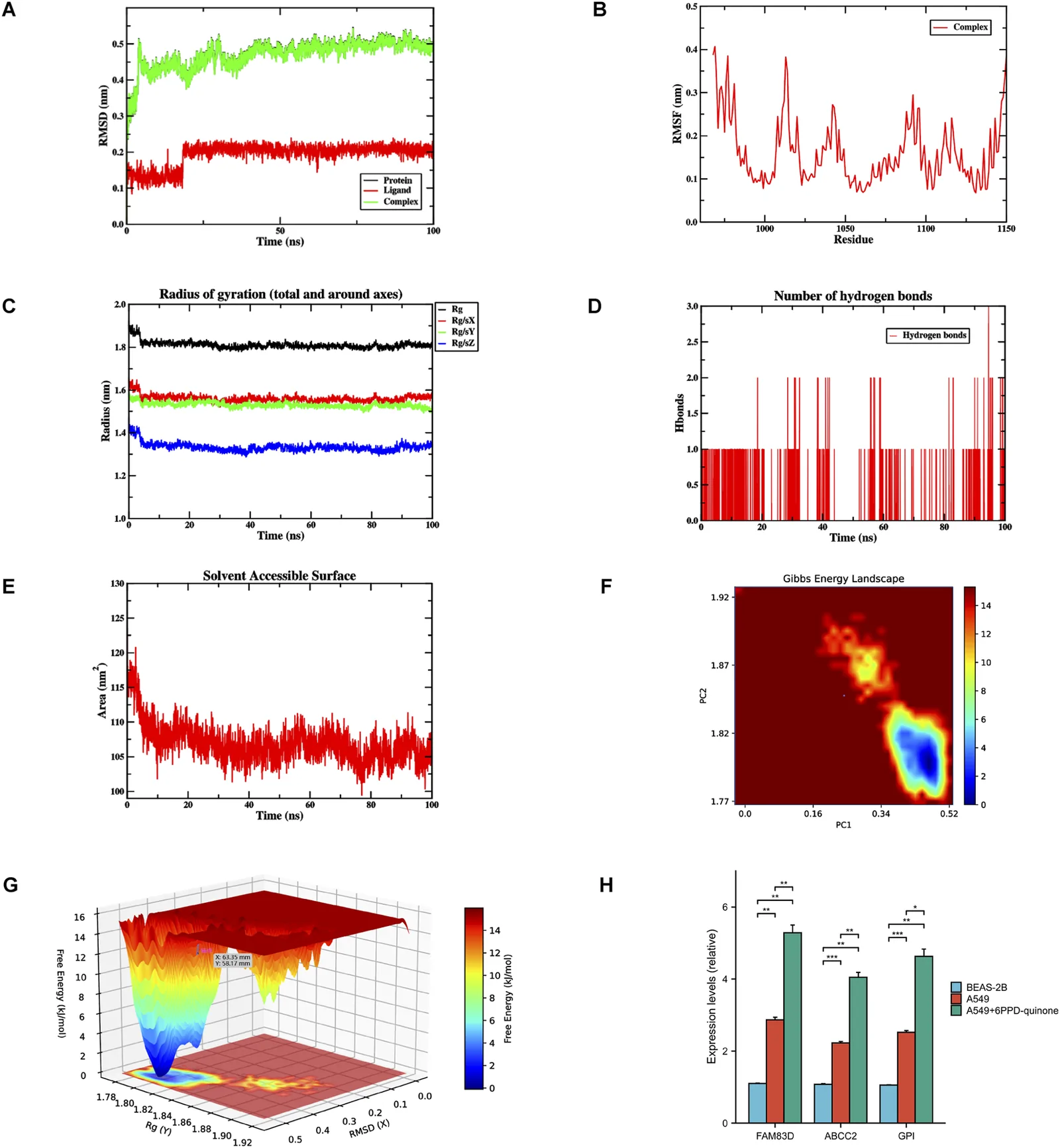
Molecular dynamics simulation results of ABCC2 binding with 6PPD-quinone. **(A)** the root-mean-square deviation. **(B)** the root-mean-square fluctuation. **(C)** the radius of gyration. **(D)** the number of hydrogen bonds. **(E)** the solvent accessible surface area. The two-dimensional **(F)** and three-dimensional **(G)** free energy landscape maps**. (H)** The mRNA expression levels of FAM83D, ABCC2, and GPI in BEAS-2B, A549, and A549 treated with 6PPD-quinone.

## Discussion

In our study, we employed an integrative network toxicology strategy combined with SHAP-based model interpretability to systemically unravel the molecular basis of 6PPD-quinone-linked LUAD through molecular docking and molecular dynamics simulations. We intersected genes related to 6PPD-quinone, those related to LUAD and the differentially expressed genes from the TCGA cohort to predict a cascade of 31 potential targets, which were further reduced to 18 network hub genes. Further prognostic modelling helped identify a seven-gene signature made up of GPI, FAM83D, KRT17, CTLA4, MAP3K8, SIX1, and ABCC2 that could reliably differentiate LUAD patients into high- and low-risk groups. Interestingly, the SHAP analysis highlighted GPI, FAM83D, KRT17, and ABCC2 as the most likely pathogenic features contributing to the model’s prediction. The mechanism behind this attribution was established via molecular docking analysis of the stable binding conformation of the 6PPD-quinone- GPI, FAM83D, and ABCC2 complex, respectively. The convergence of results indicates that hub genes may serve as a pathogenic link between chronic exposure to 6PPD-quinone and the progression of LUAD. Furthermore, these results indicate that application of a network toxicology framework could be useful in evaluating the health risks of urban air pollution.

FAM83D is a member of the FAM protein family, most members of which are closely associated with the occurrence and development of various cancers (Zhang et al., 2019; Yu et al., 2024). Shi et al. confirmed that FAM83D promotes cancer by regulating the cell cycle (Shi et al., 2016), and that invasion and migration of lung adenocarcinoma cells are significantly reduced after FAM83D knockout (Zhao et al., 2022). The oncogenic effect of FAM83D may be related to the activation of the AKT/mTOR signaling pathway (Yin et al., 2020). In addition, previous studies have shown that activating the upstream pathway of FAM83D, circFOXM1/miR-614, can also promote the proliferation and progression of non-small cell lung cancer (NSCLC) (Yu et al., 2020). ABCC2, by encoding multidrug resistance-associated protein 2 (MRP2), transports a variety of substances (including many chemotherapeutic drugs) from inside the cell to the outside, thereby enabling cancer cells to survive (Comsa et al., 2018; Shi et al., 2024; Wang et al., 2020). Studies have confirmed that METTL3 directly regulates ABCC2 expression through m6A modification (Zhang R. et al., 2024). Furthermore, knocking down ABCC2 expression can reverse the resistance of non-small cell lung cancer to chemotherapeutic drugs (Chen et al., 2020). Another study found that this drug resistance upregulates ABCC2 expression through the activation of the PFKP/NF-κB pathway (Wang et al., 2023). Moreover, ABCC2 is closely associated with lymph node metastasis in patients with lung adenocarcinoma (Li et al., 2023). Glucose-6-phosphate isomerase (GPI) was initially discovered to be expressed on the surface of eukaryotic cell membranes in an anchored manner (Ikezawa et al., 2002), functioning by modifying and retaining complement regulatory proteins, cell adhesion molecules, and receptors on the cell membrane (Murakami, 2025). In addition, GPI plays a key role in glycolysis and gluconeogenesis and is involved in cell cycle regulation in LUAD (Han et al., 2021). Recent studies have found that GPI-80 influences the growth, proliferation, and inflammation of tumor cells by regulating oxidative reactions (Takeda et al., 2021). Tang et al. revealed through constructing a lncRNA network analysis that GPI is a key gene for evaluating LUAD prognosis and chemotherapeutic response (Tang et al., 2022). Collectively, our study also indicates that FAM83D, ABCC2, and GPI, as potential pathogenic genes, may play important roles in the process of 6PPD-quinone-induced lung adenocarcinoma.

6PPD-quinone is an emerging environmental pollutant generated by the ozonation of the tire rubber antioxidant 6PPD (Chen et al., 2023). Its widespread presence in air, dust, and water bodies has raised global concern (Yi et al., 2025; Jiang et al., 2024). Although numerous studies have confirmed that 6PPD-quinone exhibits neurotoxicity, reproductive toxicity, and immunotoxicity in multiple species (Qiu et al., 2026; Wang Q.-N. et al., 2026), the molecular mechanisms linking 6PPD-quinone exposure to LUAD remain largely unexplored. Previous studies have indicated that 6PPD-quinone induces apoptosis in ovarian granulosa cells through inhibition of the PI3K/AKT/FOXO1 axis (Yu et al., 2025). Ji et al. found that high concentrations of 6PPD-quinone could be detected in the lung tissues of mice after multiple intratracheal treatments (Ji et al., 2026). Stable isotope-assisted mass spectrometry analysis revealed that after oral administration, 6PPD-quinone rapidly distributes to the lungs, with a pulmonary half-life of 20.7 ± 1.4 h, indicating significant lung retention (Zhang J. et al., 2024). This pulmonary targeting is consistent with our observation that exposure to 6PPD-quinone may drive the altered expression of lung adenocarcinoma-associated genes in lung tissue. Furthermore, existing studies have confirmed that exposure to 6PPD-quinone via the respiratory tract impairs the innate immune function of alveolar macrophages, increasing susceptibility to bacterial pneumonia (Qiu et al., 2025). It can also activate the NF-κB signaling pathway in pulmonary macrophages, triggering excessive inflammatory responses during viral infections (Qiu et al., 2026). Given that chronic inflammation is a recognized driver of carcinogenesis (Afify et al., 2022), these immunomodulatory effects may represent another mechanism by which 6PPD-quinone promotes lung adenocarcinoma development. Additionally, our findings align with known mechanisms of 6PPD-quinone toxicity, including mitochondrial dysfunction (Fang et al., 2024), oxidative stress, and immune dysregulation (Qiu et al., 2026), underscoring the plausibility of 6PPD-quinone as an environmental risk factor for lung adenocarcinoma.

The overall data suggests that 6PPD-quinone may contribute to the development of LUAD due to direct genetic dysregulation, mitochondrial dysfunction, oxidative stress, and remodeling of the immune microenvironment. As shown in tissue distribution studies, the high pulmonary retention of 6PPD-quinone strongly indicates a plausible mechanism for lung carcinogenesis. Despite the strong evidence presented in this research, there are several limitations that must be noted. To begin with, qPCR validation was done on a single concentration and time-point and the functional consequences of gene upregulation on tumorigenesis due to 6PPD-quinone needs mechanistic exploration. Also, the availability of large-scale epidemiological data linking environmental exposure to 6PPD-quinone with incidence of LUAD does not permit direct extrapolation of our findings. To determine causation and pinpoint possible alteration for environmental risk assessment, longitudinal cohort analyses and *in vivo* carcinogenesis models should be integrated in future studies that utilize hub genes, targeted therapies, or biomarkers.

## Limitations

This study presents several limitations. Firstly, molecular dynamics (MD) simulations were conducted solely for ABCC2, identified as the strongest docked binder, due to computational constraints, and the ligand charge model was not thoroughly benchmarked. Secondly, the concentration of 40 ng/mL was derived from published literature rather than our own cytotoxicity assays. Thirdly, while MAP3K8 exhibited a higher SHAP value, our selection of GPI, FAM83D, and ABCC2 as core candidates was based on a combination of evidence, including risk classification, docking affinity, and existing literature; however, this prioritization is inherently subjective. Fourthly, the proposed inflammatory mechanism is supported only by enrichment analyses and previous reports, lacking direct measurements of cytokine levels. Lastly, although our qPCR data demonstrate correlation, they do not imply causation, and we did not include BEAS 2B cells treated with 6PPD quinone in our analysis. These limitations highlight the exploratory nature of our research, indicating that future functional and mechanistic studies are necessary.

## Data Availability

The original contributions presented in the study are included in the article/Supplementary Material, Further inquiries can be directed to the corresponding authors.

## References

[B1] Afify S. M. Hassan G. Seno A. Seno M. (2022). Cancer-inducing niche: the force of chronic inflammation. Br. J. Cancer 127, 193–201. 10.1038/s41416-022-01775-w PMC9296522 35292758

[B2] Chen Y. Zhou H. Yang S. Su D. (2020). Increased ABCC2 expression predicts cisplatin resistance in non‐small cell lung cancer. Cell Biochem. Funct. 39, 277–286. 10.1002/cbf.3577 32815556 PMC7983913

[B3] Chen X. He T. Yang X. Gan Y. Qing X. Wang J. (2023). Analysis, environmental occurrence, fate and potential toxicity of tire wear compounds 6PPD and 6PPD-quinone. J. Hazard. Mater. 452, 131245. 10.1016/j.jhazmat.2023.131245 36958160

[B4] Comsa E. N. K. Loghin F. Boumendjel A. Peuchmaur M. Andrieu T. Falson P. (2018). Ovarian cancer cells cisplatin sensitization agents selected by mass cytometry target ABCC2 inhibition. Future Med. Chem. 10, 1349–1360. 10.4155/fmc-2017-0308 29848100

[B5] Damdam S. Hunnie B. Jones P. Hecker M. Brinkmann M. da Silva Junior F. C. (2026). Toxicity assessment of 6PPD-quinone in human lung cells: insights from BEAS-2B and A549 cell lines. Toxicol. Vitro 110, 106147. 10.1016/j.tiv.2025.106147 40972774

[B6] Di Federico A. Hong L. Elkrief A. Thummalapalli R. Cooper A. Ricciuti B. (2025). Lung adenocarcinomas with mucinous histology: clinical, genomic, and immune microenvironment characterization and outcomes to immunotherapy-based treatments and KRASG12C inhibitors. Ann. Oncol. 36, 297–308. 10.1016/j.annonc.2024.11.014 PMC11845285 39637943

[B7] Fang J. Wang X. Cao G. Wang F. Ru Y. Wang B. (2024). 6PPD-quinone exposure induces neuronal mitochondrial dysfunction to exacerbate Lewy neurites formation induced by α-synuclein preformed fibrils seeding. J. Hazard. Mater. 465, 133312. 10.1016/j.jhazmat.2023.133312 38147746

[B9] Han J. Deng X. Sun R. Luo M. Liang M. Gu B. (2021). GPI is a prognostic biomarker and correlates with immune infiltrates in lung adenocarcinoma. Front. Oncol. 11, 752642. 10.3389/fonc.2021.752642 PMC8666546 34912709

[B10] He W. Chao J. Gu A. Wang D. (2024). Evaluation of 6-PPD quinone toxicity on lung of male BALB/c mice by quantitative proteomics. Sci. Total Environ. 922, 171220. 10.1016/j.scitotenv.2024.171220 38412880

[B11] Hyman S. Bilić R. Jensen A. Saladin S. Tsybrii Y. Nosko O. (2025). Oxidative stress generated DNA damage by 6PPD and other tyre additives in A549 human lung epithelial cells. Sci. Rep. 15, 35615. 10.1038/s41598-025-19232-y PMC12518624 41083604

[B12] Ivanov S. M. Rudik A. V. Lagunin A. A. Filimonov D. A. Poroikov V. V. (2024). DIGEP‐Pred 2.0: a web application for predicting drug‐induced cell signaling and gene expression changes. Mol. Inf. 43, e202400032. 10.1002/minf.202400032 38979651

[B13] Ji M. Choi B. Kim I. Kim J. Park S. Hyun M. (2026). Simultaneous determination of N‐(1,3‐Dimethylbutyl)‐N′‐Phenyl‐p‐Phenylenediamine (6‐PPD) and 6‐PPD‐Quinone (6‐PPDQ) in mouse tissues after intratracheal administration using a validated LC–MS/MS method. Biomed. Chromatogr. 40, e70367. 10.1002/bmc.70367 41603216

[B14] Jiang Y. Wang C. Ma L. Gao T. Wāng Y. (2024). Environmental profiles, hazard identification, and toxicological hallmarks of emerging tire rubber-related contaminants 6PPD and 6PPD-quinone. Environ. Int. 187, 108677. 10.1016/j.envint.2024.108677 38677083

[B8] Ikezawa H. (2002). Glycosylphosphatidylinositol (GPI)-anchored proteins. Biol. Pharm. Bull. 25, 409–417. 10.1248/bpb.25.409 11995915

[B15] Kazmi S. S. U. H. Xu Q. Tayyab M. Pastorino P. Barcelò D. Yaseen Z. M. (2024). Navigating the environmental dynamics, toxicity to aquatic organisms and human associated risks of an emerging tire wear contaminant 6PPD-quinone. Environ. Pollut. 356, 124313. 10.1016/j.envpol.2024.124313 38838808

[B16] Li Q. Liu X. L. Jiang N. Li Q. Y. Song Y. X. Ke X. X. (2023). A new prognostic model for RHOV, ABCC2, and CYP4B1 to predict the prognosis and association with immune infiltration of lung adenocarcinoma. J. Thorac. Dis. 15, 1919–1934. 10.21037/jtd-23-265 PMC10183535 37197482

[B17] Li G. Li D. Zhai W. Liu C. Chen M. Xu Q. (2025). Respiratory toxicity mechanism of 6PPD and 6PPD-quinone: an integrated study based on network toxicology and molecular docking. Ecotoxicol. Environ. Saf. 301, 118494. 10.1016/j.ecoenv.2025.118494 40505274

[B18] Lin H. Hua J. Gong Z. Chen M. Qiu B. Wu Y. (2025). Multimodal radiopathological integration for prognosis and prediction of adjuvant chemotherapy benefit in resectable lung adenocarcinoma: a multicentre study. Cancer Lett. 616, 217557. 10.1016/j.canlet.2025.217557 39954935

[B19] Liu Y. Yang X. Gan J. Chen S. Xiao Z. X. Cao Y. (2022). CB-Dock2: improved protein–ligand blind docking by integrating cavity detection, docking and homologous template fitting. Nucleic Acids Res. 50, W159–W164. 10.1093/nar/gkac394 PMC9252749 35609983

[B20] Maier J. A. Martinez C. Kasavajhala K. Wickstrom L. Hauser K. E. Simmerling C. (2015). ff14SB: improving the accuracy of protein side chain and backbone parameters from ff99SB. J. Chem. Theory Comput. 11, 3696–3713. 10.1021/acs.jctc.5b00255 26574453 PMC4821407

[B21] Murakami Y. (2025). Biosynthesis of GPI anchored proteins, its deficiencies and treatment. J. Hum. Genet. 10.1038/s10038-025-01379-1 40999195

[B22] Páll S. Zhmurov A. Bauer P. Abraham M. Lundborg M. Gray A. (2020). Heterogeneous parallelization and acceleration of molecular dynamics simulations in GROMACS. J. Chem. Phys. 153, 134110. 10.1063/5.0018516 33032406

[B23] Qiu X.-W. Zhang W. Chen X. Luo G. Qi X. Guo Q. (2025). Respiratory exposure to 6PPD-quinone aggravates Klebsiella pneumoniae pneumonia by impairing the innate immune function of alveolar macrophages. J. Hazard. Mater. 496, 139277. 10.1016/j.jhazmat.2025.139277 40690842

[B24] Qiu X.-W. Chen J. Wang J. Li Y. Qi X. Zeng X. (2026). Unveiling a macrophage-driven inflammatory dysregulation: nf-κb-mediated hyperinflammation links 6PPD-Q exposure to viral immunopathology. J. Hazard. Mater. 506, 141632. 10.1016/j.jhazmat.2026.141632 41785783

[B25] Shen Y. Chen J.-Q. Li X.-P. (2025). Differences between lung adenocarcinoma and lung squamous cell carcinoma: driver genes, therapeutic targets, and clinical efficacy. Genes & Dis. 12, 101374. 10.1016/j.gendis.2024.101374 PMC11904499 40083325

[B26] Shi R. S. J. Sun Q. Zhang Q. Xia W. Dong G. Wang A. (2016). Upregulation of FAM83D promotes malignant phenotypes of lung adenocarcinoma by regulating cell cycle. Am. J. Cancer Res. 6, 2587–2598. 27904773 PMC5126275

[B27] Shi H. Tan Z. Duan B. Guo C. Li C. Luan T. (2024). LASS2 enhances chemosensitivity to cisplatin by inhibiting PP2A-mediated β-catenin dephosphorylation in a subset of stem-like bladder cancer cells. BMC Med. 22, 19. 10.1186/s12916-023-03243-5 PMC10775422 38191448

[B28] Song L. Yu X. Wu Y. Zhang W. Zhang Y. Shao Y. (2024). Integrin β8 facilitates macrophage infiltration and polarization by regulating CCL5 to promote LUAD progression. Adv. Sci. 12, 2406865. 10.1002/advs.202406865 PMC11727125 39535362

[B29] Takeda Y. Kurota Y. Kato T. Ito H. Araki A. Nara H. (2021). GPI-80 augments NF-κB activation in tumor cells. Int. J. Mol. Sci. 22, 12027. 10.3390/ijms222112027 PMC8584666 34769456

[B30] Tang W. Lu Q. Zhu J. Zheng X. Fang N. Ji S. (2022). Identification of a prognostic signature composed of GPI, IL22RA1, CCT6A and SPOCK1 for lung adenocarcinoma based on bioinformatic analysis of lncRNA-Mediated ceRNA network and sample validation. Front. Oncol. 12, 844691. 10.3389/fonc.2022.844691 35433415 PMC9012227

[B31] Tang L. Li X. Zhu S. Y. Wan Y. K. Zhang S. K. Ma J. Z. (2025). 6PPD and 6PPD quinone induce endometrial cell dysfunction *via* activating ERα and GPER at human-relevant levels. Environ. Sci. & Technol. 59, 9918–9929. 10.1021/acs.est.4c13961 40364734

[B32] Wang J.-M. Liu B. Q. Zhang Q. Hao L. Li C. Yan J. (2020). ISG15 suppresses translation of ABCC2 via ISGylation of hnRNPA2B1 and enhances drug sensitivity in cisplatin resistant ovarian cancer cells. Biochimica Biophysica Acta (BBA) - Mol. Cell Res. 1867, 118647. 10.1016/j.bbamcr.2020.118647 31926942

[B33] Wang Z. Wu S. Chen X. Liang J. Xue F. Cho W. C. (2023). PFKP confers chemoresistance by upregulating ABCC2 transporter in non-small cell lung cancer. Transl. Lung Cancer Res. 12, 2294–2309. 10.21037/tlcr-23-567 38090515 PMC10713276

[B34] Wang Z. Hu Y. Zhu M. Yang X. Huang Q. Zhang A. (2026a). The AhR/ROS-mediated lipid peroxidation pathway contributes to 6PPDQ-induced intestine-specific injury in zebrafish during embryonic development. Fish & Shellfish Immunol. 173, 111268. 10.1016/j.fsi.2026.111268 41819393

[B35] Wang Q.-N. Wang C. Wāng Y. (2026b). Inhalation exposure to tire rubber particle-sourced pollutant 6PPD-quinone involving basolateral amygdala impairment in male ICR mice. J. Adv. Res. 79, 293–311. 10.1016/j.jare.2025.03.033 PMC12766199 40118340

[B36] Xiong X. Liu W. Yao C. (2025). Development of an alkaliptosis-related lncRNA risk model and immunotherapy target analysis in lung adenocarcinoma. Front. Genet. 16, 1573480. 10.3389/fgene.2025.1573480 40264452 PMC12011837

[B37] Yang X. Liu Y. Gan J. Xiao Z.-X. Cao Y. (2022). FitDock: protein–ligand docking by template fitting. Briefings Bioinforma. 23, bbac087. 10.1093/bib/bbac087 35289358

[B38] Yao K. Kang Q. Liu W. Chen D. Wang L. Li S. (2024). Chronic exposure to tire rubber-derived contaminant 6PPD-quinone impairs sperm quality and induces the damage of reproductive capacity in male mice. J. Hazard. Mater. 470, 134165. 10.1016/j.jhazmat.2024.134165 38574660

[B39] Yi J. Ruan J. Yu H. Wu B. Zhao J. Wang H. (2025). Environmental fate, toxicity, and mitigation of 6PPD and 6PPD-Quinone: current understanding and future directions. Environ. Pollut. 375, 126352. 10.1016/j.envpol.2025.126352 40316240

[B40] Yin C. Lin X. Wang Y. Liu X. Xiao Y. Liu J. (2020). FAM83D promotes epithelial-mesenchymal transition, invasion and cisplatin resistance through regulating the AKT/mTOR pathway in non-small-cell lung cancer. Cell. Oncol. 43, 395–407. 10.1007/s13402-020-00494-9 32006253 PMC12990745

[B41] Yu C. Cheng Z. Cui S. Mao X. Li B. Fu Y. (2020). circFOXM1 promotes proliferation of non-small cell lung carcinoma cells by acting as a ceRNA to upregulate FAM83D. J. Exp. & Clin. Cancer Res. 39, 55. 10.1186/s13046-020-01555-5 32228656 PMC7106704

[B42] Yu X. Li Y. Kong F. Xu Q. (2024). METTL3 regulates FAM83D m6A modification to accelerate tumorigenesis of triple-negative breast cancer via the Wnt/β-catenin pathway. Toxicol. Vitro 95, 105746. 10.1016/j.tiv.2023.105746 38043628

[B43] Yu H. Zhang W. Wang D. Shi B. Zhu Y. Hu W. (2025). Exposure to 6PPD-Q induces dysfunctions of ovarian granulosa cells: its potential role in PCOS. J. Hazard. Mater. 486, 137037. 10.1016/j.jhazmat.2024.137037 39764971

[B44] Zhang Q. Yu S. Lok S. I. S. Wong A. S. T. Jiao Y. Lee L. T. O. (2019). FAM83D promotes ovarian cancer progression and its potential application in diagnosis of invasive ovarian cancer. J. Cell. Mol. Med. 23, 4569–4581. 10.1111/jcmm.14360 31037837 PMC6584551

[B45] Zhang R. Chen P. Wang Y. Zeng Z. Yang H. Li M. (2024a). Targeting METTL3 enhances the chemosensitivity of non-small cell lung cancer cells by decreasing ABCC2 expression in an m6A-YTHDF1-dependent manner. Int. J. Biol. Sci. 20, 4750–4766. 10.7150/ijbs.97425 PMC11414383 39309428

[B46] Zhang J. Cao G. Wang W. Qiao H. Chen Y. Wang X. (2024b). Stable isotope-assisted mass spectrometry reveals *in vivo* distribution, metabolism, and excretion of tire rubber-derived 6PPD-quinone in mice. Sci. Total Environ. 912, 169291. 10.1016/j.scitotenv.2023.169291 38104817

[B47] Zhang P. Wang D. Zhou G. Jiang S. Zhang G. Zhang L. (2025). Novel post-translational modification learning signature reveals B4GALT2 as an immune exclusion regulator in lung adenocarcinoma. J. Immunother. Cancer 13, e010787. 10.1136/jitc-2024-010787 40010763 PMC11865799

[B48] Zhao T. Wang M. Zhao X. Weng S. Qian K. Shi K. (2022). YTHDF2 inhibits the migration and invasion of lung adenocarcinoma by negatively regulating the FAM83D-TGFβ1-SMAD2/3 pathway. Front. Oncol. 12, 763341. 10.3389/fonc.2022.763341 35186724 PMC8847186

[B49] Zhao H. N. Thomas S. P. Zylka M. J. Dorrestein P. C. Hu W. (2023). Urine excretion, organ distribution, and placental transfer of 6PPD and 6PPD-Quinone in mice and potential developmental toxicity through nuclear receptor pathways. Environ. Sci. & Technol. 57, 13429–13438. 10.1021/acs.est.3c05026 PMC11648498 37642336

[B50] Zhu X. Xu Y. (2025). Gut microbiome contributes to 6PPD-quinone induced cognitive impairment through PI3K/Akt signaling. Toxicology 517, 154217. 10.1016/j.tox.2025.154217 40505719

[B51] Zuo X. Hou X. Tan S. Zhang C. Zhang Y. Sun R. (2026). Network toxicology and multi-omics analyses identify diagnostic genes and elucidate underlying mechanisms of 6PPDQ-induced hepatocellular carcinoma. Environ. Pollut. 391, 127632. 10.1016/j.envpol.2026.127632 41485723

