# Supplementary material for: Identification of potential pathogenic genes for lung adenocarcinoma induced by 6PPD-quinone through network toxicology and SHAP analysis
Source: Front Toxicol. 2026 Oct 19;8:1948595. doi: 10.3389/ftox.2026.1948595 (PMC13724384; doi:10.3389/ftox.2026.1948595)
Supplement: Supplementary file 1 [file Supplementaryfile1.docx]

**FIGURE S1** | The Venn diagram displays the intersecting genes (brown) among Comparative Toxicogenomics Databases (red), GeneCards (blue), and OMIM (orange).
