# Supplementary material for: Identification of potential pathogenic genes for lung adenocarcinoma induced by 6PPD-quinone through network toxicology and SHAP analysis
Source: Front Toxicol. 2026 Oct 19;8:1948595. doi: 10.3389/ftox.2026.1948595 (PMC13724384; doi:10.3389/ftox.2026.1948595)
Supplement: Supplementary file 2 [file Table1.docx]

**Supplementary Table S1** | Sequences of primers used in the study.

| Gene | Forward(5’-3’) | Reverse(5’-3’) |
| --- | --- | --- |
| FAM83D | GCGTGGCAACAGGCTCCTACA | GGCATACAGGATTCGGAACTCC |
| ABCC2 | GCCAACTTGTGGCTGTGATAGG | ATCCAGGACTGCTGTGGGACAT |
| GPI | CTGGTAGACGGCAAGGATGTGA | TCCGTGATGGTCTTGCCTGTGT |
| GAPDH | GGAGCGAGATCCCTCCAAAAT | GGCTGTTGTCATACTTCTCATGG |
